# Three *Orobanche* genomes reveal distinct molecular repertoires in species with different host ranges

**DOI:** 10.1093/pcp/pcaf047

**Published:** 2025-05-18

**Authors:** Marco Bürger, Charmaine F Soco, Miguel A García, Yoshiya Seto, Antonio Leyva

**Affiliations:** Plant Biology Laboratory, Salk Institute for Biological Studies, 10010 North Torrey Pines Road, La Jolla, CA 92037, USA; Howard Hughes Medical Institute, Salk Institute for Biological Studies, 10010 North Torrey Pines Road, La Jolla, CA 92037, USA; Division of Hematology, Oncology, Stem Cell Transplantation and Regenerative Medicine, Center for Definitive and Curative Medicine, Department of Pediatrics, Stanford University School of Medicine, 300 Pasteur Dr, Palo Alto, CA 94304, USA; Real Jardín Botánico, Consejo Superior de Investigaciones Científicas, Pl. Murillo, 2, Retiro, 28014 Madrid, Spain; Laboratory of Plant Chemical Regulation, School of Agriculture, Meiji University, 1-1-1, Higashi-mita, Tama-ku, Kawasaki 214-8571, Japan; Department of Plant Molecular Genetics, Centro Nacional de Biotecnología, Consejo Superior de Investigaciones Científicas, C. Darwin, 3, Fuencarral-El Pardo, 28049 Madrid, Spain

**Keywords:** parasitic plants, *Orobanche*, whole-genome sequencing, genome evolution, strigolactones

## Abstract

Parasitic plants in the genus *Orobanche* display varying levels of host specificity, however, the molecular features determining host range are not fully understood. We sequenced the genomes of three *Orobanche* species with different host ranges: *Orobanche minor* (generalist), *O. gracilis* (*Fabaceae*-specific), and *O. hederae* (ivy-specific). Comparative analysis revealed considerable genome size variation but consistent holoparasitic gene loss patterns. While synteny analysis revealed strong collinearity between *O. gracilis*, *O. hederae*, and *O. minor*, we found distinct molecular repertoires among species. *O. gracilis* showed expanded protein-degrading and carbohydrate-active enzyme families, while putative strigolactone receptor (KAI2d) numbers varied from five in specialists to eleven in the generalist *O. minor*, suggesting diverse molecular strategies across different host ranges.

## Introduction

Parasitic plants have evolved multiple times independently across the plant kingdom, with one of the most specialized forms being holoparasitism, where plants have lost their photosynthetic capacity and rely entirely on their hosts for nutrients (Westwood et al. 2010). The lifestyle of root-parasitic plants is facilitated by strigolactones (SLs), plant hormones that are exuded by host roots. SLs exist in various structural variations, including host-specific non-canonical SLs (Bürger and Chory 2020). Parasitic plants have evolved to detect these different strigolactone molecules through the diversification of KAI2d proteins, which act as strigolactone receptors (Conn et al. 2015, Nelson 2021). The number of these receptors varies between parasitic plant species, potentially reflecting their adaptation to different host ranges. The genus *Orobanche* (broomrapes) represents an intriguing group of holoparasitic plants that has diversified to parasitize a wide range of host species. Within this genus, the relationship between host range and evolutionary adaptation is often poorly understood, particularly regarding the genomic basis of host specificity versus generalism (Thorogood and Hiscock 2010, Hatt et al. 2024). A compelling example of this evolutionary puzzle can be found in the comparison between *O. minor* and *Orobanche hederae*. These species are morphologically quite similar and are frequently confused in the field (Thorogood and Rumsey 2020), yet they exhibit strikingly different host ranges. While *O. minor* is a generalist capable of parasitizing numerous host species across multiple plant families, *O. hederae* is highly specialized, parasitizing predominantly *Araliaceae*, usually *Hedera* sp. (Fernández-Aparicio et al. 2009). This contrast raises fundamental questions about the genomic architecture underlying host specialization and the evolutionary trajectories leading to such divergent strategies while maintaining morphological similarity. *Orobanche gracilis* represents a more divergent member of the genus, with distinct morphological features and exclusively parasitizing members of the *Fabaceae* family (Román et al. 2007). While it shows preference for *Ulex* and *Genista* species, it can also parasitize other legumes such as *Lotus corniculatus* (common bird’s-foot trefoil) and *Hippocrepis comosa* (horseshoe vetch). The inclusion of this species provides a broader perspective on the evolution of parasitism within *Orobanche* and allows us to investigate whether similar genomic changes underlie different instances of host specialization. Recent advances in genomic resources for parasitic plants, including the published genomes of *Orobanche cumana* (Xu et al. 2022) and *Orobanche coerulescens* (Kim et al. 2024), have provided insights into the genetic basis of parasitism in this genus. However, questions about the evolution of host range and specialization remain. Here, we present the genome sequences of *O. minor*, *O. hederae*, and *O. gracilis*, and through comparative analysis with previously published *Orobanche* genomes, we investigate the genomic features associated with different parasitic lifestyles and host ranges within this genus.

## Results

### *Orobanche* genomes vary in size but maintain typical holoparasitic gene loss

**Figure 1:**
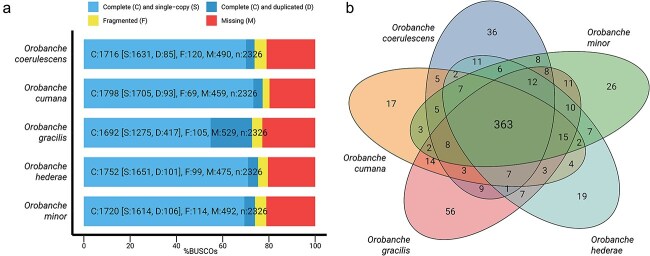
BUSCO completeness assessment and analysis of missing genes across five *Orobanche* species. (a) BUSCO assessment results using the eudicots_odb10 dataset (*n* = 2326). Bars show the proportion of complete single-copy (light blue), complete duplicated (dark blue), fragmented (yellow), and missing (red) BUSCO genes. Numbers above bars indicate the exact count for each category. (b) Venn diagram showing the distribution and overlap of missing BUSCO genes among species. Numbers in each section represent the count of BUSCOs missing in the corresponding species or species combinations. The central number (363) represents BUSCOs missing in all five species.

**Figure 2:**
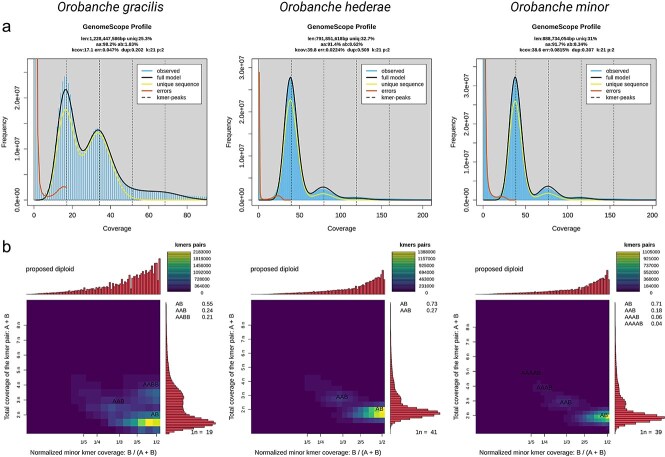
Genome characteristics of three *Orobanche* species based on k-mer analysis. (a) GenomeScope profiles showing k-mer frequency distributions of sequencing reads. Blue histograms represent observed k-mer frequencies, black lines show the fitted model, orange lines indicate sequencing errors, and dashed vertical lines mark coverage peaks. GenomeScope metrics are: len: estimated haploid genome size; uniq: unique portion of the genome; kcov: mean k-mer coverage; dup: genome duplication level; het: heterozygosity (%). (b) Smudgeplots depicting genome ploidy patterns. Heatmaps show the total k-mer coverage versus normalized minor k-mer coverage, with color intensity indicating k-mer pair frequency. Side panels show k-mer pair distributions and ploidy estimates based on AB, AAB, and AABB values.

We sequenced the genomes of *O. gracilis*, *O. hederae*, and *O. minor*, and compared them to the published genomes of *O. coerulescens* and *O. cumana*. While *O. coerulescens* and *O. cumana* are available as chromosome-level assemblies, our new assemblies show high contiguity with scaffold N50 values ranging from 31.5 Mb (*O. hederae*) to 104.3 Mb (*O. minor*). *O. minor*’s assembly comprises 556 scaffolds with the largest being 210.2 Mb, while *O. gracilis* and *O. hederae* assemblies are more compact with 314 and 311 scaffolds respectively (Table 1). The assembled genome sizes varied considerably among the five *Orobanche* species, ranging from 1.46 Gb in *O. cumana* to 3.65 Gb in *O. coerulescens*, while GC content remained relatively constant across all species (40.56%–41.05%). The number of predicted protein-coding genes (CDS) ranged from 25 141 in *O. cumana* to 35 001 in *O. minor*, with *O. gracilis* and *O. coerulescens* also showing high numbers (34 306 and 34 245, respectively). The number of unique proteins (proteins lacking detectable orthologs in all other genomes using the following thresholds: *E*-value ≤1e−5, ≥25% sequence identity, and ≥50% alignment coverage) varied more, with *O. coerulescens* having the highest number (11 961) followed by *O. minor* (9719), while *O. cumana* and *O. hederae* showed considerably fewer unique proteins (5421 and 5474, respectively). Benchmarking Universal Single-Copy Orthologs (BUSCO) analysis (Manni et al. 2021) revealed similar levels of completeness across most species (72.7%–77.3%), with *O. cumana* showing the highest percentage of complete BUSCOs (77.3%). Notably, *O. gracilis* displayed a distinctive pattern with a substantially higher proportion of duplicated BUSCOs (17.9%) compared to other species (3.7%–4.6%), while having the lowest percentage of single-copy BUSCOs (54.8%). The proportion of missing BUSCOs was relatively consistent across all species, ranging from 19.7% in *O. cumana* to 22.8% in *O. gracilis*, which is expected from the genomes of holoparasitic plants (Fig. 1a). Further analysis of missing BUSCO genes revealed both species-specific and shared patterns of gene loss across the five *Orobanche* species. *O. gracilis* showed the highest number of missing BUSCOs (529), followed by *O. minor* (493), *O. coerulescens* (491), *O. hederae* (476), and *O. cumana* (460). A substantial core set of 363 BUSCOs was consistently missing across all five species, suggesting these genes are dispensable for the holoparasitic lifestyle. Pairwise comparisons showed the highest number of shared missing genes between *O. gracilis* and *O. minor* (429 BUSCOs), while *O. coerulescens* and *O. cumana* shared the fewest (400 BUSCOs). Each species also displayed a set of uniquely missing BUSCOs, with *O. gracilis* showing the largest number of species-specific losses (Fig. 1b). To investigate whether the observed differences in genome size and gene content might be attributed to variations in ploidy levels, we performed k-mer analysis of sequencing reads using GenomeScope (Fig. 2a) in conjunction with Smudgeplot (Fig. 2b; Ranallo-Benavidez et al. 2020), which suggested that our newly sequenced genomes are diploid, consistent with the diploidy of the previously published genomes of *O. coerulescens* and *O. cumana*.

**Table 1:** Genome assembly statistics and BUSCO assessment of five *Orobanche* species.

	*Orobanche coerulescens* Kim et al. (2024)	*Orobanche cumana* Xu et al. (2022)	*Orobanche gracilis* (This study)	*Orobanche hederae* (This study)	*Orobanche **minor*** (This study)
Assembly size (bp)	3 648 003 138	1 463 172 293	2 070 951 698	2 373 151 697	2 541 775 564
Num scaffolds	Chromosome-level assembly	Chromosome-level assembly	314	311	556
Scaffold N50 (bp)			41 083 608	31 491 747	104 325 404
Percent GC	41.04	40.65	41.05	40.68	40.56
Number of CDS	34 245	25 141	34 306	27 636	35 001
Unique proteins	11 961	5421	8292	5474	9719
Complete BUSCOs	73.8%	77.3%	72.7%	75.3%	74.0%
Single-copy BUSCOs	70.1%	73.3%	54.8%	71.0%	69.4%
Duplicated BUSCOs	3.7%	4.0%	17.9%	4.3%	4.6%
Fragmented BUSCOs	5.2%	3.0%	4.5%	4.3%	4.9%
Missing BUSCOs	21.0%	19.7%	22.8%	20.4%	21.1%

The table shows genome size, GC content, predicted genes (CDS), unique proteins (proteins lacking detectable orthologs in all other genomes using the following thresholds: *E*-value ≤1e−5, ≥25% sequence identity, and ≥50% alignment coverage), and BUSCO completeness metrics. BUSCO analysis was performed using the eudicot dataset (eudicots_odb10) as reference. CDS: coding sequences.

### Genome synteny reveals high collinearity between *O. gracilis*, *O. hederae*, and *O. minor*

**Figure 3:**
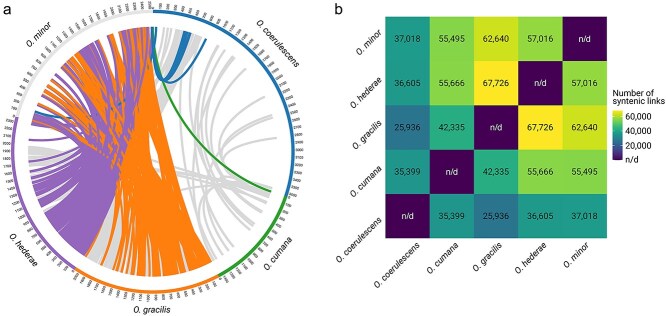
Visualization of syntenic relationships between five *Orobanche* species. (a) Circos plot showing the top 1000 syntenic links between the genomes, limited for visualization purposes. Each segment on the circle represents a species with its scaled genome size. Colored ribbons indicate syntenic regions between species (purple: links between *O. hederae* and *O. minor*; orange: links between *O. gracilis* and *O. minor*; green: links between *O. cumana* and *O. minor*; blue: links between *O. coerulescens* and *O. minor*; grey: remaining links). (b) Heatmap showing the total number of whole-genome syntenic links between species pairs. Color intensity indicates the number of syntenic relationships, with yellow representing higher numbers of links. Values in each cell show the exact number of syntenic links between species pairs, with “n/d” indicating the diagonal where self-comparisons are not determined.

To obtain insights into the collinear relationships between the five examined *Orobanche* species we carried out a whole-genome synteny analysis. The highest number of syntenic links was observed between *O. gracilis* and *O. hederae* (67 726 links), followed by *O. gracilis* and *O. minor* (62 640 links). *O. hederae* and *O. minor* shared 57 016 syntenic regions, while *O. cumana* showed similar numbers of syntenic relationships with both *O. hederae* (55 666 links) and *O. minor* (55 495 links). *O. coerulescens* exhibited relatively fewer syntenic relationships, ranging from 25 936 links with *O. gracilis* to 37 018 links with *O. minor* (Fig. 3). This pattern suggests varying degrees of genome conservation between these species, with particularly strong syntenic preservation between *O. gracilis*, *O. hederae*, and *O. minor*.

### Genome-wide and protein-specific analyses confirm close relationship between *O. minor* and *O. hederae*

**Figure 4:**
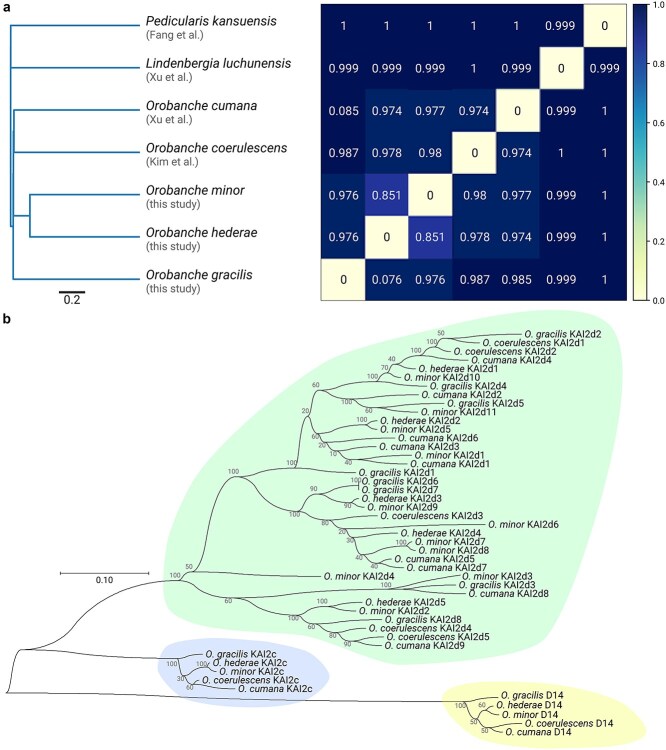
Phylogenetic relationships between *Orobanche* species based on genome-wide and protein-specific analyses. (a) Genome-wide distance matrix based on k-mer comparison using sourmash. The heatmap shows pairwise distances between five *Orobanche* species and two outgroups. Darker blue indicates greater genomic distance, while lighter colors indicate higher sequence similarity. (b) Maximum likelihood phylogenetic tree of D14 and KAI2 proteins from five *Orobanche* species. The tree shows three distinct clades (highlighted in yellow, blue, and green): the conserved D14 clade, the KAI2c clade, and the expanded KAI2d clade. Bootstrap support values are shown at the nodes.

We assessed genome-wide sequence similarities between the five *Orobanche* species and two outgroup species [*Lindenbergia luchunensis* (Xu et al. 2022) and *Pedicularis kansuensis* (Fang et al. 2024)] using sourmash, a k-mer based comparison method (Irber et al. 2024). The resulting distance matrix revealed that within *Orobanche*, *O. minor* and *O. hederae* showed the highest sequence similarity (distance = 0.851), while *O. gracilis* and *O. coerulescens* were more divergent (distance = 0.987). The two outgroup species showed expected high distances from all *Orobanche* species, with distances approaching the maximum value of 1.0 (Fig. 4a). The phylogenetic tree constructed from these distances placed *O. minor* and *O. hederae* as sister species, forming a well-defined clade. *O. cumana*, *O. coerulescens*, and *O. gracilis* formed successive branching points in the phylogeny. Both *L. luchunensis* and *P. kansuensis* were positioned as clear outgroups, consistent with their taxonomic classification outside the genus *Orobanche*. These distance-matrix based results are in agreement with a previous ITS and trnL-trnF based phylogenetic study focused on *Orobanche* species that has placed *O. minor* and *O. hederae* as sister species and *O. cumana* and *O. coerulescens* in a group that is distinct from *O. gracilis* (Piwowarczyk et al. 2021).

Analysis of key signaling proteins further supported these genome-wide relationships. We constructed a phylogenetic tree using D14, KAI2c, and KAI2d protein sequences from the investigated *Orobanche* species. In the more conserved D14 and KAI2c clades, *O. minor* and *O. hederae* proteins clustered together, with *O. gracilis* sequences showing the greatest divergence. This pattern was similar in the faster-evolving KAI2d clade, where despite overall higher sequence divergence, *O. minor* and *O. hederae* KAI2d proteins maintained their close evolutionary relationship. These protein-specific phylogenetic patterns align with the genome-wide k-mer based analysis, providing additional support for the close evolutionary relationship between *O. minor* and *O. hederae* (Fig. 4b).

### *O. gracilis* shows expanded repertoire of protein-degrading and carbohydrate-active enzymes

**Figure 5:**
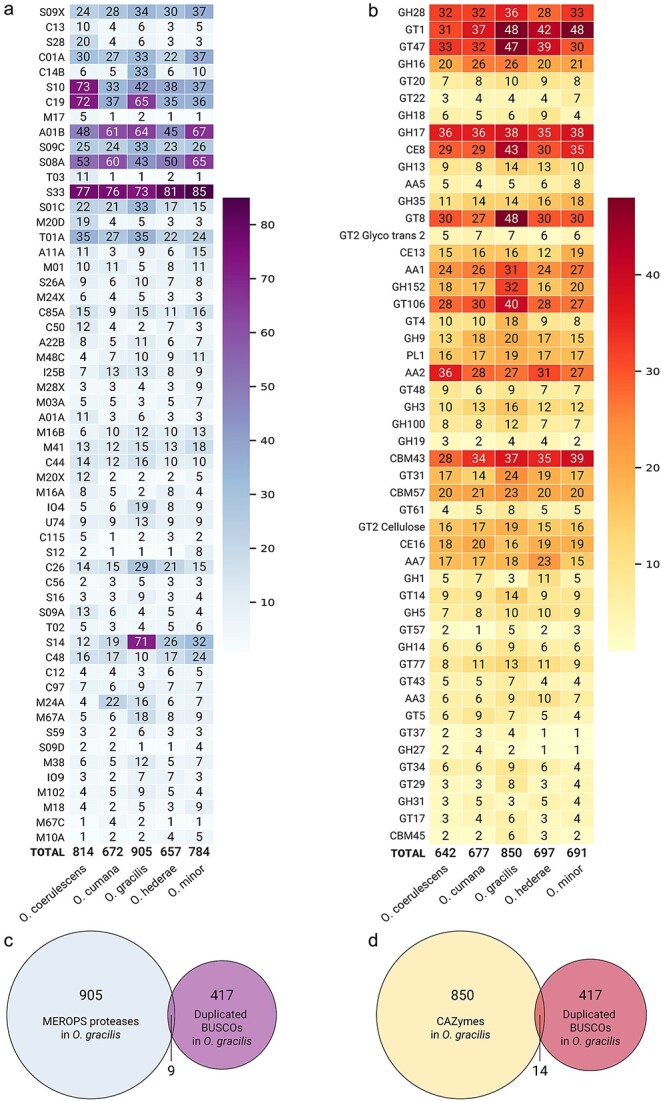
Distribution of protein-degrading and carbohydrate-active enzymes across *Orobanche* species identified using funannotate compare. (a) Heatmap showing the abundance of MEROPS peptidase families in five *Orobanche* species. The color intensity (white to purple) represents the number of genes in each family, with darker purple indicating higher abundance. (b) Heatmap displaying the abundance of CAZyme families. Color intensity (yellow to dark red) indicates the number of genes in each family. (c) Venn diagram showing the overlap between duplicated BUSCOs and MEROPS proteases in *O. gracilis*. (d) Venn diagram showing the overlap between duplicated BUSCOs and CAZymes in *O. gracilis*.

To assess the diversity and distribution of protein-degrading enzymes and carbohydrate-active enzymes across *Orobanche* species, we analyzed the abundance of peptidase database (MEROPS) and Carbohydrate-Active enZYmes (CAZyme) genes in each genome. Serine proteases (S) represented the largest group in all species, ranging from 302 members in *O. cumana* to 388 in *O. gracilis*, followed by cysteine proteases (C) with 161–246 members, and metalloproteases (M) with 124–169 members. *O. gracilis* and *O. minor* showed the highest overall protease counts, with particularly expanded families of serine (388 and 350 members, respectively) and cysteine proteases (246 and 191 members). Within individual MEROPS families, the serine protease family S33 was consistently the most abundant across all species (73–85 members). The second most abundant family varied between species: C19 in *O. coerulescens* (72 members) and *O. gracilis* (65 members), while *O. cumana*, *O. hederae*, and *O. minor* showed high numbers of A01B aspartic proteases (61, 45, and 67 members, respectively). Some protease families showed species-specific expansions, such as the S10 family in *O. coerulescens* (73 members) and the S14 family in *O. gracilis* (71 members). In contrast, *O. hederae* displayed generally lower numbers across most protease classes, though maintaining similar proportions among the different catalytic types. Protease inhibitors (I) showed notable variation, with *O. gracilis* containing 45 members compared to 21–30 in other species (Fig. 5a). Analysis of carbohydrate-active enzymes (CAZymes) revealed distinct glycoside-processing profiles across *Orobanche* species. Glycosyltransferases (GT) and glycoside hydrolases (GH) were the most abundant CAZyme classes in all species, with GT ranging from 281 members in *O. coerulescens* to 411 in *O. gracilis*, and GH ranging from 229 to 291 members. *O. gracilis* showed notably higher numbers of both GT and GH enzymes compared to other species. Auxiliary activities (AA), carbohydrate esterases (CE), and carbohydrate-binding modules (CBM) were present in moderate numbers (56–99 members), while polysaccharide lyases (PL) were consistently low across all species (23–25 members). Among individual CAZyme families, GT1 was particularly abundant in most species (37–48 members), especially in *O. gracilis* and *O. minor* (48 members each). The GH17 family showed high conservation across species (35–38 members). Some species showed unique patterns, such as *O. coerulescens* having equal numbers of GH17 and AA2 (36 members each), while *O. gracilis* uniquely showed high numbers of GT8 (48 members) and GT47 (47 members). *O. minor* and *O. cumana* shared elevated numbers of CBM43 (39 and 34 members, respectively; Fig. 5b). To investigate whether the expanded enzyme repertoire in *O. gracilis* might be attributed to its higher proportion of duplicated BUSCOs, we analyzed the overlap between duplicated BUSCOs and enzyme families. We found only minimal overlap, with just 9 duplicated BUSCOs among the 905 MEROPS peptidases (Fig. 5c) and 14 duplicated BUSCOs among the 850 CAZymes (Fig. 5d), indicating that the enzyme family expansions in *O. gracilis* are largely independent of BUSCO gene duplications.

### Functional analysis reveals diverse molecular adaptations across *Orobanche* genomes

**Figure 6:**
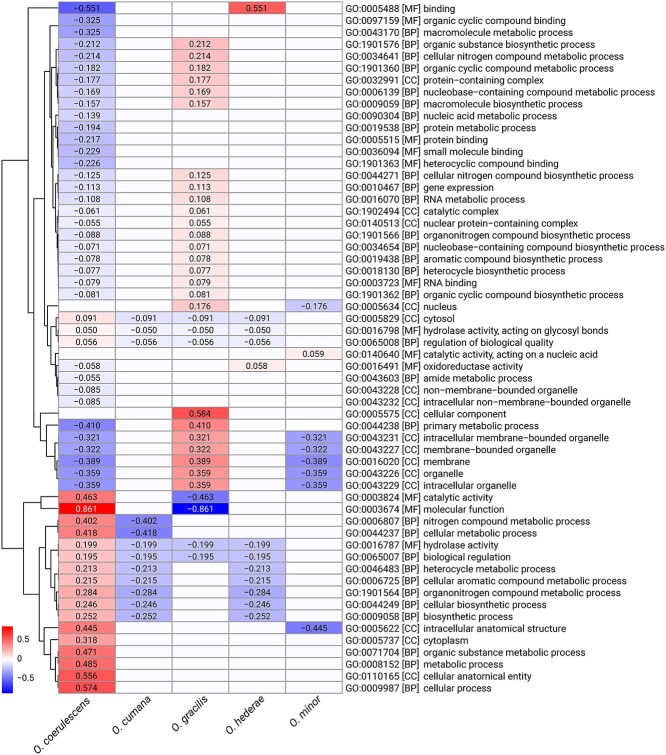
Gene Ontology enrichment analysis across five *Orobanche* species. Heatmap showing significantly enriched or depleted GO terms (absolute enrichment value ≥0.05). Blue indicates underrepresentation and red indicates overrepresentation of GO terms. Each row represents a GO term with its identifier and description, and each column represents a species. Values in cells indicate the enrichment score, with empty cells representing non-significant enrichment (|value| < 0.05). Rows are clustered based on enrichment patterns across species using Euclidean distance and complete linkage clustering. GO categories are indicated in square brackets: MF (molecular function), BP (biological process), and CC (cellular component).

To identify potential functional adaptations and differences between the *Orobanche* species, we performed Gene Ontology (GO) enrichment analysis across the five genomes. *O. coerulescens* showed the most distinctive profile, with significant overrepresentation of molecular function terms and cellular process-related terms. In contrast, *O. gracilis* displayed significant underrepresentation in molecular function categories but showed strong enrichment in cellular component terms. Notably, binding-related GO terms showed contrasting patterns between species, being significantly depleted in *O. coerulescens* but enriched in *O. minor*. Both *O. hederae* and *O. minor* exhibited similar patterns in several metabolic process categories, including nucleobase-containing compound metabolic process and cellular nitrogen compound metabolic process, suggesting functional conservation between these closely related species. Interestingly, membrane-associated terms and organelle-related categories showed variable enrichment patterns across species, with *O. gracilis* and *O. minor* displaying notable differences in these cellular component terms (Fig. 6).

## Discussion

Here, we present the genome sequences of *O. minor*, *O. gracilis*, and *O. hederae*. Our comparative analysis of all five available *Orobanche* genomes reveals important insights into genome evolution and potential adaptations related to parasitism and host specificity. We observed substantial variation in genome sizes (1.46–3.65 Gb) yet consistent pattern of BUSCO gene loss across all species (19.8%–22.8% missing). The variation in genome sizes despite similar gene loss patterns suggests that non-coding DNA elements are responsible for the observed size differences, which is in agreement with a previous study demonstrating that repetitive DNAs appear to be major contributors to genome size variation among *Orobanchaceae* (Piednoël et al. 2012). While our k-mer analysis suggests that all studied *Orobanche* samples are diploid, this does not preclude the possibility of ancient whole-genome duplications (WGDs) followed by diploidization in the evolutionary history of these parasitic plants. Such ancient WGDs could contribute to the observed variation in genome sizes and gene content, particularly the elevated duplication rates observed in *O. gracilis*. Chromosome counts have suggested that *O. gracilis* is tetraploid, with some hexaploid examples (Weiss-Schneeweiss et al. 2006). These prior samples, however, did not include the population that we sequenced here, and we plan to sample the population used for this study for chromosome counting in the future.

The diversification of kai2d genes is considered a major driver of parasitism in the *Orobanchaceae* (Conn et al. 2015). *O. minor*, a generalist capable of parasitizing hosts across multiple plant families, possesses the highest number of KAI2d receptors (11, including previously unidentified KAI2d6-11 in addition to the known KAI2d1-5), suggesting that receptor diversification may facilitate the recognition of various host-derived signals. This aligns with our understanding that KAI2d proteins can detect both strigolactones and sesquiterpene lactones, potentially enabling *O. minor* to respond to a broader spectrum of host-derived chemicals (Takei et al. 2023). In contrast, the ivy-specialist *O. hederae* maintains only five KAI2d receptors (with one likely non-functional due to sequence truncation), and *O. coerulescens*, which specifically parasitizes *Artemisia* species, also has five receptors. This reduced receptor repertoire in specialists might reflect adaptation to specific host-derived signals. *O. gracilis*, which occupies an intermediate position by specifically parasitizing members of the *Fabaceae* family, maintains eight KAI2d receptors, possibly reflecting the need to detect varied germination signals across different legume genera. *O. cumana*, despite its specialization on sunflower, harbors nine KAI2d receptors (Xu et al. 2022). We speculate that this expanded receptor family might be linked to its highly virulent nature, potentially enabling detection of various germination stimulants produced by its agriculturally important host, and at least one of these receptors has been shown to perceive dehydrocostus lactone as well (Han et al. 2024). It has been demonstrated that, in general, higher virulence in *O. cumana* is associated with greater genetic diversity (Martín-Sanz et al. 2016). Beyond germination, protein-degrading and carbohydrate-active enzymes play an essential role in host invasion (Singh and Singh 1993, Losner-Goshen 1998, Ogawa et al. 2021). The expanded repertoire of these enzymes in *O. gracilis*, particularly its higher numbers of GT and GH enzymes, may reflect adaptations to its specific host range within the *Fabaceae*. The substantial expansion of GT8 and GT47 families, which are often involved in cell wall modification (Brown et al. 2005, Zhong et al. 2005, Sterling et al. 2006, Persson et al. 2007), could be related to specialized invasion strategies required for legume parasitism. Similarly, the high proportion of duplicated BUSCOs (17.9%) in *O. gracilis* suggests that gene duplication may have facilitated its adaptation to legume hosts. Our findings also highlight *O. coerulescens* as particularly distinct, showing the lowest syntenic conservation with other species and a unique GO term enrichment profile. This divergence might reflect its separate evolutionary history or adaptation to different hosts, though further research is needed to understand the functional implications of these differences. Although there seems to be no correlation between assembly quality and duplication patterns, our analyses are based on single genome assemblies for each *Orobanche* species, which precludes statistical analysis of intraspecific variation. The observed genomic patterns, including the high proportion of duplicated BUSCOs and expansion of enzyme families in *O. gracilis* compared to other species, represent single data points that may not capture the full range of variation within each species. Subsequent studies incorporating multiple individuals per species would be valuable to establish the consistency of these patterns across populations and to statistically assess the relationship between genomic features and host specificity.

These results provide a foundation for understanding the genomic basis of host range evolution in parasitic plants. The contrast between the *O. minor* and *O. hederae* genomes shows that transitions between generalist and specialist strategies likely involve substantial genomic changes that extend beyond the evolution of strigolactone receptor genes. While these species maintain high synteny, the considerable differences in the number of coding sequences (35 001 in *O. minor* versus 27 636 in *O. hederae*) and unique proteins (9719 versus 5474, respectively) suggest broader genomic adaptations accompanying host specialization. Our comparative analysis of five *Orobanche* species demonstrates that host specialization is shaped by genomic changes that include substantial gene loss, diversification of strigolactone receptors, and species-specific enzyme family expansions. Future studies focusing on gene regulation and protein function will be crucial for understanding how these genomic differences exactly translate into host specificity.

## Materials and Methods

### Sample collection

*Orobanche hederae* was collected from a naturalized and reliable population at coordinates 37.8714218° N, −122.2653102° W, adjacent to the University of California, Berkeley campus on 20 May 2024, at an elevation of 67 m, where it grows parasitically on a patch of *Hedera algeriensis* on a well-maintained lawn under a *Quercus agrifolia* tree. Several voucher specimens for this population are available at the Jepson Herbarium (JEPS), including JEPS121581, JEPS126696, and JEPS135848. *O. gracilis* was collected from a known and reliable population at coordinates 40.7115283° N, −3.6062033° W, near San Agustín de Guadalix in the Comunidad de Madrid, Spain, on 11 May 2021, at an elevation of 772 m. The habitat, a transitional zone between montane scrubland and lowland meadow, consisted of dry, semi-arid grassland with sparse vegetation, including scattered shrubs or subshrubs such as *Retama sphaerocarpa*, *Cistus albidus*, *Pistacia terebinthus*, *Colutea arborescens*, *Rhamnus alaternus*, *Rhamnus lycioides*, *Thymus* spp., *Helichrysum stoechas*, *Psoralea bituminosa*, *Phagnalon saxatile*, *Mercurialis tomentosa*, *Ruta montana*; small trees, such as *Quercus ilex*, *Quercus coccifera*, and *Juniperus oxycedrus.* We assume that *R. sphaerocarpa* is the host because *O. gracilis* is associated with this legume in this population. A voucher specimen is conserved at the Real Jardín Botánico herbarium (MA) under MA-01-00966823. *O. minor* was cultivated in a rhizotron system within the laboratory in April 2022, using *Trifolium pratense* as the host plant. The seeds were sourced from the Nagasawa Water Filtration Plant (coordinates 35.6067214° N, 139.5437315° E). A voucher specimen of this naturalized population is available under NAC-VA195322 at the Nagasawa Environmental Conservation Research Institute (NAC).

### Genomic DNA isolation

Genomic DNA was isolated using the Nanobind plant nuclei kit (PacBio). Between 5 and 30 μg of genomic DNA was isolated from around 1 g of flower tissue from an individual plant of each species.

### Genome sequencing and assembly

Genomic DNA was first quantified on a Qubit 3.0 and sized using a Genomic ScreenTape on an Agilent TapeStation 4200. Samples meeting QC guidelines (50% >30 kb) were selected for library preparation. DNA was sheared using a Covaris g-Tube and centrifuged at 2750 rpm in an Eppendorf 5430R to target 13–20 kb fragments. The DNA underwent four full passes prior to cleanup with SMRTbell cleanup beads. This purified DNA was then used to generate SMRTbell libraries using the PacBio SMRTbell 3.0 kit following manufacturer’s protocol. Libraries were size-selected using 35% Ampure PB beads to remove fragments <5 kb. Final libraries were quantified using a Qubit 3.0 and sized using an Agilent Femto Pulse with a Genomic DNA 165 kb kit. Libraries were loaded onto the PacBio Revio at a targeted loading concentration of 200–300 pM per sample. HiFi reads were generated on the PacBio Revio system using 25 m SMRT cells in 30-h sequencing runs, with base calling and quality filtering performed on-instrument using SMRT Link v13.0.0 and CCS v8.0.0 (Pacific Biosciences). Sequencing was conducted on the PacBio Revio system for 30 h per sample. The raw HiFi reads were assembled into contigs using Hifiasm v0.24.0 (Cheng et al. 2021). Default parameters were used for the assembly, and quality control metrics were calculated using assembly-stats v1.0.1 (Sanger Pathogen 2016). For *O. gracilis*, a single run yielded 61.08 Gb of HiFi reads with a median read quality of Q38. The HiFi reads had a mean length of 16 299 bp and a median length of 15 427 bp. From the sequencing run, 3 831 598 ZMWs passed filters (50.13% of input), resulting in 3 747 270 HiFi reads. Of the total bases sequenced, 94.4% (57.67 Gb) had quality scores ≥Q30. The coverage depth was 19×. For *O. hederae*, two runs yielded a combined total of 93.73 Gb of HiFi reads with a median read quality of Q45.5 (averaged from Q45 and Q46). The HiFi reads had a mean length of 7469 bp and a median length of 6032 bp (averaged from the two runs). From the sequencing runs, 12 777 804 ZMWs passed filters (43.34% of input), resulting in 12 544 297 HiFi reads. Of the total bases sequenced, 96.4% (90.35 Gb) had quality scores ≥Q30. The coverage depth was 41×. For *O. minor*, two runs yielded a combined total of 98.35 Gb of HiFi reads with a median read quality of Q38.5 (averaged from Q38 and Q39). The HiFi reads had a mean length of 13 061 bp and a median length of 12 641 bp (averaged from the two runs). From the sequencing runs, 7 524 583 ZMWs passed filters (35.03% of input), resulting in 7 524 901 HiFi reads. Of the total bases sequenced, 95.15% (93.59 Gb) had quality scores ≥Q30. The coverage depth was 39×.

### Gene prediction and annotation

*Orobanche* gene prediction was performed using Helixer v0.3.4 (Stiehler et al. 2021) and functional annotation was carried out using InterProScan v5.59-91.0 (Jones et al. 2014) and EggNOG-mapper v2.1.12 (Cantalapiedra et al. 2021).

### BUSCO assessment and analysis of missing genes

Genome completeness was assessed using BUSCO v4.1.2 (Manni et al. 2021) with the eudicots_odb10 lineage dataset. To identify shared patterns of gene loss, missing BUSCO groups were extracted from the BUSCO output files and analyzed for overlaps between species using the R package VennDiagram (Chen 2022).

### Synteny analysis

Genome-wide alignments were performed using minimap2 v2.28 (Li 2021) with default parameters to identify syntenic regions between species pairs. The resulting alignments were converted to Circos links format and visualized using Circos v0.69.6 (Krzywinski et al. 2009). Additionally, a quantitative representation of syntenic relationships was generated as a heatmap using R packages ggplot2 (Wickham 2016), reshape2 (Wickham 2007), and viridis (Garnier et al. 2023).

### Phylogenetic analysis

Genome-wide phylogenetic relationships were assessed using sourmash v3.3.0 (Irber et al. 2024) to generate a k-mer based distance matrix and tree. For protein-specific phylogenetic analysis, D14 and KAI2 protein sequences were aligned and analyzed using MEGA11 (Tamura et al. 2021), with maximum likelihood trees constructed under default parameters. To identify D14 and KAI2 candidate proteins, BLAST databases were first created from the protein FASTA files generated by the genome annotation pipeline. BLASTp searches were conducted against these databases using *Arabidopsis thaliana* DWARF14 as query and applying following thresholds: *E*-value ≤ 0.0001, sequence identity ≥25%, and alignment length ≥220 amino acids. Candidate protein sequences were then extracted, and duplicate sequences were removed.

### Enzyme and GO term analysis

Distribution of protein-degrading enzymes and carbohydrate-active enzymes across *Orobanche* species was determined using funannotate v1.7.4 (Palmer and Stajich 2023). GO enrichment analysis was performed across the entire proteomes using the same tool, employing Fisher’s exact test with Benjamini–Hochberg FDR correction (threshold FDR < 0.05) to identify significantly enriched or depleted GO terms. The resulting GO enrichment values were filtered to retain terms with absolute enrichment values ≥0.05. The filtered dataset was visualized as a heatmap using the R packages dplyr (H. Wickham et al. 2023a), pheatmap (Kolde 2019), and tidyr (H. Wickham et al. 2023b), with hierarchical clustering of GO terms based on Euclidean distances and complete linkage method.

## Data Availability

The genome sequences, gene models, and annotations of *Orobanche gracilis*, *O. hederae*, and *O. minor* have been deposited in the NCBI GenBank under BioProject PRJNA1230823 and their accession numbers JBLZII000000000, JBLZIJ000000000, and JBLZIK000000000, respectively.
